# Combined BET bromodomain and CDK2 inhibition in MYC-driven medulloblastoma

**DOI:** 10.1038/s41388-018-0135-1

**Published:** 2018-03-07

**Authors:** Sara Bolin, Anna Borgenvik, Camilla U. Persson, Anders Sundström, Jun Qi, James E. Bradner, William A. Weiss, Yoon-Jae Cho, Holger Weishaupt, Fredrik J. Swartling

**Affiliations:** 1 Department of Immunology, Genetics and Pathology, Science for Life Laboratory, Rudbeck Laboratory, Uppsala University, Uppsala, Sweden; 2 Department of Medicine, Harvard Medical School; Department of Medical Oncology, Dana-Farber Cancer Institute, Boston, MA USA; 3 Departments of Neurology, Pediatrics and Neurosurgery, University of California, San Francisco, CA USA; 4 Department of Pediatrics, Papé Family Pediatric Research Institute, Knight Cancer Institute, Oregon Health & Science University, Portland, OR USA

## Abstract

Medulloblastoma (MB) is the most common malignant brain tumor in children. MYC genes are frequently amplified and correlate with poor prognosis in MB. BET bromodomains recognize acetylated lysine residues and often promote and maintain MYC transcription. Certain cyclin-dependent kinases (CDKs) are further known to support MYC stabilization in tumor cells. In this report, MB cells were suppressed by combined targeting of MYC expression and MYC stabilization using BET bromodomain inhibition and CDK2 inhibition, respectively. Such combination treatment worked synergistically and caused cell cycle arrest as well as massive apoptosis. Immediate transcriptional changes from this combined MYC blockade were found using RNA-Seq profiling and showed remarkable similarities to changes in MYC target gene expression when MYCN was turned off with doxycycline in our MYCN-inducible animal model for Group 3 MB. In addition, the combination treatment significantly prolonged survival as compared to single-agent therapy in orthotopically transplanted human Group 3 MB with MYC amplifications. Our data suggest that dual inhibition of CDK2 and BET bromodomains can be a novel treatment approach for suppressing MYC-driven cancer.

## Introduction

Medulloblastoma (MB) is the most common malignant pediatric brain tumor [1]. Current therapies of MB improve patient survival by about 70% and include surgical resection, radiation therapy, and chemotherapy [2]. MB pathogenesis implies an early embryonic initiating aberration in a number of important developmental genes that predispose children to MB. Gene expression profiling divides MB into four molecularly distinct subgroups including Wingless (WNT), Sonic Hedgehog (SHH), Group 3, and Group 4 [3]. MYC genes, most commonly *MYC* and *MYCN*, are frequently amplified in MB [4] and are associated with a poor prognosis [5] and/or tumor recurrence [6].

Transcription factors, like MYC proteins, are poor therapeutic targets [7] with short half-lives and pleiotropic natures. Recent alternative strategies allow epigenetic regulation of MYC transcription and MYC target genes through inhibition of bromodomain and extraterminal (BET)-containing proteins. BET-containing proteins recognize acetylated lysine residues on euchromatin and promote transcription [8]. MYC genes and their transcriptional output have demonstrated to be quite specific targets in cancer [9]. Additionally, BET inhibition has most recently been shown to be a potential novel therapeutic strategy for MYC-amplified MB patients [10, 11] and MYCN-amplified neuroblastoma patients [12].

We previously used two different MB models to show that brain tumors became addicted to the MYCN oncogene and that MYCN stabilization was required for MB development [13, 14]. These models are useful tools in drug screens aiming to identify specific therapeutic approaches to treat MYCN-driven cancers. Cyclin-dependent kinases (CDK), especially CDK1 and CDK2, are key players in stabilizing phosphorylation of MYC proteins at Serine-62 upon activation [15–17]. CDKs are dependent on cyclins for their activity and these complexes play an important role in regulating the progression of the cell cycle. Consequently, CDKs and various cyclins are often upregulated in cancer cells, including MB [18–20]. CDK suppression using the PAN CDK-inhibitor Purvalanol A is effective in targeting MYC-driven tumors in vitro but cannot alone suppress tumor growth in MYC-overexpressing transgenic animals [21]. Interestingly, specific CDK2 inhibition is found to be synthetically lethal to MYCN-driven neuroblastoma [22] suggesting a potential role for CDK2-inhibiting drugs also in MB-carrying amplifications in MYCN and perhaps also MYC.

Group 3 MB often presents itself with elevated MYC overexpression or MYC amplifications and has the worst prognosis of the four MB groups with <50% survival [23]. By contrast, MYCN amplifications are more common in SHH tumors and the largest molecular subgroup of Group 4 tumors. Given the heavy radio and chemotherapy offered to high-risk patients today with subsequent side effects including severe neurocognitive defects [24], it is imperative to find novel, more targeted therapeutic strategies with fewer side effects. By testing a set of specific CDK inhibitors alone or together with BET bromodomain inhibition, we propose a need for combined targeting in order to more effectively treat aggressive MYC/MYCN-driven MB.

## Results

### CDK and BET bromodomain inhibitors target MYC proteins

**Fig. 1:**
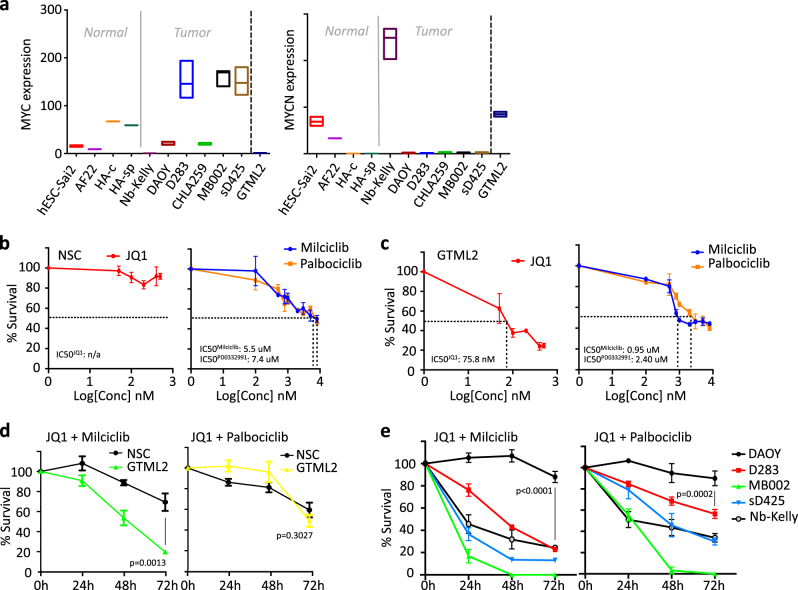
MYC/MYCN-amplified tumors are particularly sensitive to BET and CDK inhibition. **a** Expression of MYC and MYCN in normal and MB cells based on RNA sequencing. Dose–response curves of **b** normal NSC and **c** GTML2 treated with 0–500 nM JQ1, 0–15 μM Milciclib, or 0–15 μM Palbociclib. **d** Survival of murine NSCs and GTML2 cells with combination treatment using JQ1 and CDK inhibitors (JQ1 and Milciclib 500 nM; Palbociclib 2 μM). **e** Survival of human DAOY, D283, sD425 (MB004), and MB002 MB cells and a neuroblastoma cell line Kelly with combination treatment using JQ1 and CDK inhibitors (JQ1 and Milciclib 500 nM; Palbociclib 2 μM)

We used RNA-Seq to select human MB cell lines that showed high levels of MYC gene expression in order to see if they would be suitable for transcriptional cross-species comparisons with our Glt1-tTA:TRE-MYCN/Luc (GTML) murine MB Group 3 cell lines. We selected GTML2 cells derived from isolated MB biopsies of transgenic GTML mice where human wild-type MYCN can be effectively turned off by giving doxycycline (DOX) [13]. RNA-Seq expression levels (fragments per kilobase of exon per million fragments mapped (FPKM)) showed high levels of MYC in human Group 3 MB lines D283, sD425, and MB002 (Fig. 1a) and high levels of MYCN in GTML2 cells and in a human neuroblastoma cell line, Kelly, that was also included in the analysis. We previously used single treatments of JQ1 in MB cells that showed good efficacy in inhibiting GTML2 MB cells induced by MYCN [14] as compared to normal neural stem cell (NSC) controls isolated from newborn cerebellum (Supplementary Figure 1a). As CDK inhibition is also likely to inhibit MYC levels, we tested a broad CDK inhibitor, Purvalanol A, that targets not only CDK1 and CDK2 but also CDK4 [25]. Purvalanol indeed reduced the overall survival of murine MB cells after 72 h of treatment (*p* < 0.0005). However, control NSCs responded equally well and were almost equally efficiently eradicated by Purvalanol A treatment (Supplementary Figure 1b). As this strategy might be general in killing any dividing cells, it would be worth studying a more restricted CDK inhibition approach to see if we could also get a more targeted inhibition of tumor cells. Dose–response curves (Fig. 1b, c) of normal NSCs and GTML2 cells following a 72 h treatment with BET bromodomain inhibitor JQ1, selective CDK2 inhibitor Milciclib [26], selective CDK4/6 inhibitor Palbociclib [27], or doxycycline (DOX) (Supplementary Figure 1c) showed an increased response to increasing concentration of the active compound. NSCs were less affected by JQ1 treatment. Not even a high concentration of 1500 nM JQ1 (data not shown in graph) generated cell death with any measurable IC50 values (Fig. 1b). GTML2 cells responded well to JQ1 with an IC50 of 75.8 nM (Fig. 1c). Indeed, selective CDK2 or CDK4/6 inhibition showed less efficacy as compared to Purvalanol in targeting MB cells and NSCs. However, CDK2 inhibition with Milciclib was targeting MB cells more effectively than normal NSCs at IC50 0.95 μM compared to 5.5 μM, respectively (Fig. 1b, c). Further, as compared to DOX-treated GTML2 cells, increasing concentrations of the individual compounds cannot completely eliminate the tumor cells (Fig. 1b; Supplementary Figure 1c). Interestingly, JQ1 in combination with Milciclib or Palbociclib resulted in effective cell death of brain tumor cells. However, while Purvalanol alone killed 75% of all normal NSCs after 72 h, JQ1 with Milciclib or Palbociclib combination treatment only killed 30 and 42% of the normal NSCs, respectively (Fig. 1d). Moreover, the JQ1 and Milciclib combination both earlier and more effectively reduced the cell viability of the MYC-amplified Group 3 MB cell lines MB002, sD425, and D283 and the MYCN-amplified neuroblastoma cell line Kelly, as compared to the JQ1 and Palbociclib combination (Fig. 1e). Interestingly, while MYC-amplified MB cells responded to single treatment (Supplementary Figures 1d,e), the non-MYC-amplified MB line DAOY neither responded to JQ1, Milciclib, or Palbociclib nor the combinations of JQ1 together with these specific CDK inhibitors (Fig. 1e, Supplementary Figure 1f).

### Combination treatment leads to MYC suppression and promotes apoptosis

**Fig. 2:**
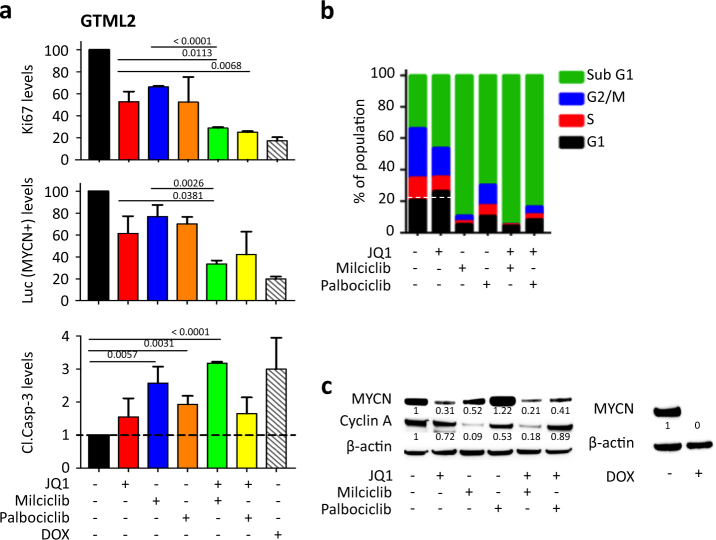
Combined BET and CDK inhibition leads to cell cycle blockade, increased MYCN inhibition, and massive apoptosis. **a** Quantification of intracellular staining of Ki67, Luciferase, and cleaved caspase-3 positive in GTML2 after indicated 72 h treatment. Fluorescent signal was analyzed using a BD LSR II multi-laser analytical flow cytometer, BD Biosciences. **b** Cell cycle analysis of GTML2 after 72 h of single or combination treatments using JQ1, Milciclib, and Palbociclib alone or in combination. **c** Western blot of MYCN and cyclin A protein levels in GTML2 cells after 72 h challenge with single or combinatorial treatment. Statistical analysis: Student's *t*-test

Next we asked how the MYC-targeted treatment is causing cell death by looking at markers for proliferation, cell death, and suppression of the MYC or MYCN protein itself. JQ1 together with the selective CDK inhibitors reduced the levels of proliferative marker Ki67 and luciferase (MYCN) and increased cleaved caspase-3 activity compared to controls in GTML2 (Fig. 2a, Supplementary Figure 2a) after 72 h treatment. BET inhibitors are known to cause G1-S arrest in many tumors while CDK inhibitors including Milciclib similarly cause apoptosis [28–30]. We similarly saw that JQ1 halted cells in G1 while combination treatment drastically increased the sub-G1 population (Fig. 2b; Supplementary Figure 2b). The cell cycle profile of normal NSCs showed reduced changes after treatment compared to GTML2 (Supplementary Figure 2c). Treatment with JQ1 alone or JQ1 together with Milciclib or Palbociclib reduced MYCN protein levels below the relative ratio of 0.5 as compared to the control (1.0) in GTML2 cells after 72 h (Fig. 2c). JQ1 treatment in combination with Milciclib further reduced MYCN levels as compared to JQ1 treatment alone. However, MYCN was not regulated at the transcriptional level following any treatment. Instead, mRNA levels of MYCN slightly increased after 72 h treatment as compared to dimethyl sulfoxide (DMSO)-treated controls (Supplementary Figure 2d). As expected, inhibition of CDK2 with Milciclib alone or in combination with JQ1 selectively reduced Cyclin A levels in GTML2 compared to control (Fig. 2c), in line with results from Milciclib treatment in GBM cells [31]. To summarize, cell death and cell cycle arrest was most prominently affected and inhibited by Milciclib alone or in combination with JQ1. By comparison, Palbociclib treatment alone or in combination with JQ1 was not as efficient as Milciclib (Fig. 2a, b) and Purvalanol treatment. MYCN levels were also most effectively suppressed by Milciclib, especially in combination with JQ1 (Fig. 2c). Since Palbociclib treatment only suppressed MYCN in combination with JQ1, we decided from now on to focus on Milciclib alone or in combination with JQ1 for targeting MYC and MYCN-driven brain tumors.

### Immediate transcriptome effects of BET bromodomain and CDK2 treatment

**Fig. 3:**
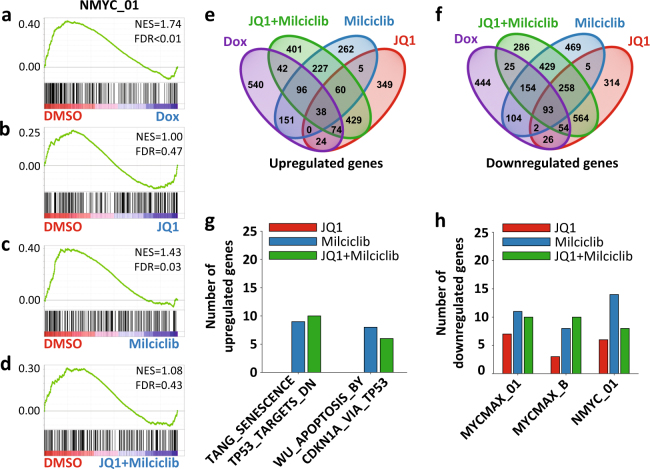
Comparing the transcriptional output from MYCN suppression with BET and CDK inhibition in order to identify essential gene targets. Characterizing the transcriptional changes induced after 6 h treatments. **a**–**d** GSEA results for the top MYCN target gene-related gene set (NMYC_01) downregulated in GTML-DOX-6h as compared to GTML–DMSO-6h (**a**); the GSEAs for the same gene set are shown comparing GTML-DMSO-6h with GTML-JQ1-6h (**b**), GTML-Milciclib-6h (**c**), and GTML-JQ1+Milciclib-6h (**d**). Enrichments were considered significant if FDR < 0.05. **e**, **f** Venn diagrams displaying the number of genes significantly upregulated (**e**) or downregulated (**f**) in each treatment as compared to DMSO and the overlap of regulated genes between treatments. **g**, **h** Bar plot depicting the number of genes significantly upregulated in two TP53/apoptosis gene sets (**g**) or downregulated in three MYC/MYCN gene sets (**h**) in GTML-DOX-6h as compared to GTML-DMSO-6h and shared with similarly regulated genes in GTML-JQ1-6h, GTML-Milciclib-6h, or GTML-JQ1+Milciclib-6h; gene sets were identified in **a** and Supplementary Figure 3b. Genes were considered significantly regulated if at least one condition was expressed (log10(FPKM + 1) > 0.50), the FDR adjusted *p*-value *q* < 0.05, and if log2(FC) > log2(1.5) (upregulated) or log2(FC) < −log2(1.5) (downregulated)

To identify genes directly involved in MYC-dependent MB cell death, we used RNA-Seq to study the immediate transcriptional changes when turning off MYCN in our DOX-inducible GTML MB model. We compared DMSO-treated GTML2 cells (GTML-DMSO) and cells treated with DOX for 6 h (GTML-DOX) using a targeted gene set enrichment analysis (GSEA) on a selection of four MYCN-related gene sets and observed a significant (fals discovery rate (FDR) < 0.01) downregulation of putative MYCN transcription factor binding sites in the DOX-treated cells (Fig. 3a). A subsequent GSEA between GTML-DMSO and GTML-JQ1 (Fig. 3b, FDR = 0.47), GTML-Milciclib (Fig. 3c, FDR = 0.03), or GTML-JQ1+Milciclib (Fig. 3d, FDR = 0.43), respectively, indicated a significant downregulation (at a FDR significance threshold of *α* = 0.05) of these target genes only in Milciclib treatment.

JQ1 has been described as a potential drug for suppressing the output of MYC/MYCN-driven transcription [10] but the role of Milciclib in suppressing MYC/MYCN levels is not known. While DOX treatment suppressed MYCN mRNA levels already after 6 h, JQ1, Milciclib, or the combination of JQ1 and Milciclib did not show any immediate suppression of MYCN at the transcriptional level (Supplementary Figure 3a). We next aimed to determine additional and advantageous effects contributed by the Milciclib treatment. For this purpose, we performed unbiased GSEAs on four databases between GTML-DMSO and GTML-DOX, GTML-JQ1, GTML-Milciclib, or GTML-JQ1+Milciclib (Supplementary Table 1). Among the gene sets that were significantly enriched and regulated in the same way in GTML-DOX and GTML-Milciclib but not in GTML-JQ1 were two downregulated sets of MYC target genes and two upregulated sets of TP53/apoptosis gene sets (Supplementary Figure 3b). To investigate in more detail how such MYC/MYCN and TP53/apoptosis target genes with differential expression between GTML-DMSO and GTML-DOX were affected by JQ1 and Milciclib treatments, we identified genes significantly regulated in the same fashion between different treatments (Fig. 3e, f). Comparing genes upregulated in GTML-DOX and GTML-JQ1, GTML-DOX and GTML-Milciclib, or GTML-DOX and GTML-JQ1+Milciclib indicated that the combination treatment upregulated more TP53 and apoptosis genes as compared to the JQ1 treatment, with the major contribution originating from the Milciclib treatment (Fig. 3g). Similarly, the combination treatment downregulated more of the investigated MYC/MYCN target genes as compared to the JQ1 treatment alone, while the number of downregulated genes was highest in the Milciclib treatment (Fig. 3h). Together, these findings suggest that Milciclib contributed at least in two ways, achieving an increased upregulation of apoptotic signatures and downregulation of MYC/MYCN signatures in the combination treatment as compared to the JQ1 treatment alone.

Finally, to evaluate the performance of the JQ1 and Milciclib treatments on suppressing more general MB subgroup signatures, we performed GSEAs against a MB Group 3 and a MB Group 4 signature gene set (Supplementary Figure 3c). Milciclib appeared to be more effective (FDR = 0.02) as compared to JQ1 (FDR = 0.37) at downregulating Group 4 signature genes. While a comparison of the normalized enrichment score values hinted at an opposite trend for the MB Group 3 signature, with JQ1 better suited to suppress these genes, none of the enrichments passed the FDR significance threshold of FDR < 0.05. However, the combination treatment achieved a significant (FDR < 0.05) downregulation of MB Group 3 genes.

**Fig. 4:**
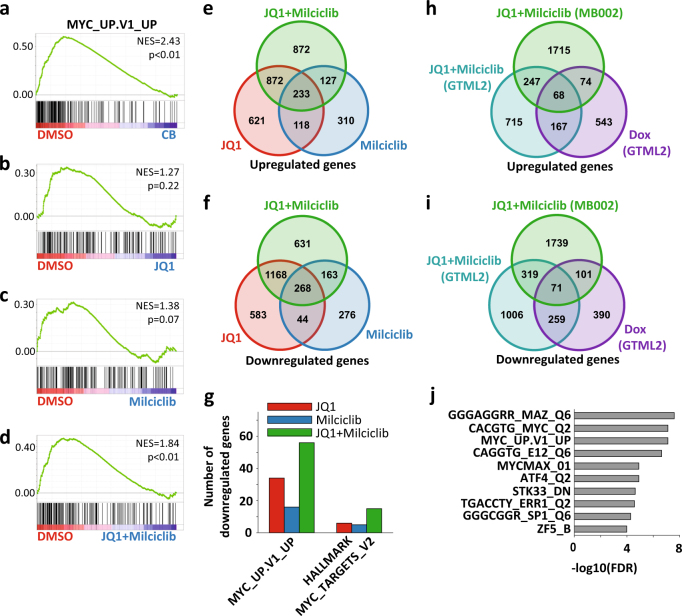
Combined BET and CDK2 inhibition targets the MYC transcriptional output in MYC-amplified human medulloblastoma. **a**–**d** GSEA result for the oncogenic signature-related gene set (MYC_UP.V1_UP) most strongly downregulated in cerebellar cells (**a**) and MB002-JQ1+Milciclib-6h (**d**) as compared to MB002-DMSO-6h; the GSEAs for the same gene set are shown comparing MB002-DMSO-6h with MB002-JQ1-6h (**b**) and MB002-Milciclib-6h (**c**). Enrichments were considered significant if FDR < 0.05. **e**, **f** Venn diagrams displaying the number of genes significantly upregulated (**e**) or significantly downregulated (**f**) in each treatment as compared to DMSO and the overlap of regulated genes between treatments. **g** Bar plot depicting the number of genes significantly downregulated in two MYC target gene-related gene sets in GTML-JQ1-6h, GTML-Milciclib-6h, or GTML-JQ1+Milciclib-6h; gene sets were identified in **a** and Supplementary Figure 4a. **h**, **i** Venn diagrams showing the number of genes upregulated (**h**) or downregulated (**i**) in GTML-DOX-6h, GTML-JQ1+Milciclib-6h, and MB002-JQ1 + Milciclib-6h as compared to GTML-DMSO-6h and the overlap of regulated genes between treatments. Genes were considered significantly regulated if at least one condition was expressed, i.e., log10(FPKM + 1) > 0.50 (GTML) or log10(FPKM + 1) > 0.60 (MB002), the FDR-adjusted *p*-value *q* < 0.05, and if log2(FC) > log2(1.5) (upregulated) or log2(FC) < −log2(1.5) (downregulated). **j** The ten gene sets with top enrichment according to a GSO analysis of the shared 71 downregulated genes from **i**

In order to check whether the inhibition of human MYC-amplified tumors involved similar transcriptional mechanisms as the DOX-treated murine MYCN-driven tumors, we used RNA-Seq analysis on MB002 cells treated with the two drugs alone or in combination and normal cerebellum total RNA as normal control. An initial GSEA comparing MB002 DMSO-treated cells (MB002-DMSO) to cerebellar control cells on four databases of gene sets (Supplementary Table 2a) revealed genes putatively upregulated by MYC as the most strongly downregulated oncogenic signature gene set in cerebellar cells (Fig. 4a, FDR < 0.05). Interestingly, the gene set was not significantly regulated in either MB002-JQ1 (Fig. 4b, FDR = 0.22) or MB002-Milciclib (Fig. 4c, FDR = 0.07) but significantly regulated in MB002-JQ1+Milciclib (Fig. 4d, FDR < 0.05) using a FDR significance threshold of *α* = 0.05. Consistent with the contribution of Milciclib described in Fig. 3 however, the GSEA also identified another MYC-related gene set with significant downregulation in cerebellar cells, Milciclib, and combination treatment but not in JQ1 treatment (Supplementary Figure 4a).

Again, we investigated these gene sets in the context of differential expression and started by identifying differentially expressed genes overlapping between the different treatments (Fig. 4e, f). Consistent with the previous observations, the combination treatment achieved a downregulation of more MYC target genes as compared to either of the single treatments (Fig. 4g), again emphasizing the potential benefit of combining these drugs. In agreement with the results on the GTML2 cells, JQ1 also performed better than Milciclib in downregulating MB Group 3 signature genes in MB002 cells (Supplementary Figure 4b). In summary, in human MB002 cells, JQ1 and Milciclib seemed to have complementary effects for downregulated MYC responses, while JQ1 showed a better effect at suppressing MB-related signature genes.

Finally, in order to obtain a more robust set of genes and functions regulated by the combination treatment, we integrated the results of the RNA-Seq on GTML2 and MB002 cells. Specifically, assuming the DOX treatment of GTML2 cells as the ideal treatment, we identified genes that were significantly regulated in the same fashion in GTML-DOX, GTML-JQ1+Milciclib, and MB002-JQ1+Milciclib, resulting in 59 genes commonly upregulated (Fig. 4h) and 71 genes commonly downregulated (Fig. 4i, Supplementary Table 2b). Interestingly, in a gene set overlap (GSO) analysis of the 71 downregulated genes against four different gene set databases, at least three of the top ten most significant gene sets were related to MYC/MYCN target genes (Fig. 4j), thus demonstrating a clear potential of the combination treatment in downregulating the transcriptional output of MYC proteins. Among downregulated genes, typical MYC target genes including USP2 [32] and JAG2 could be found. The deubiquitinating enzyme USP2 has been found to enhance MYC levels through the modulation of specific subsets of microRNAs in prostate cancer [32]. Further, the expression of the NOTCH ligand JAG2 can be induced by MYC-induced transcriptional activation and the expression of JAG2 and MYC correlate well in Group 3 MB [33]. Interestingly, USP2 significantly correlated with poor survival in Group 3 MB patients when analyzing patients with high as compared to low USP2 mRNA levels in a cohort of tumors from 113 patients [34] (Supplementary Figure 4c). A similar trend was seen for JAG2 in where elevated JAG2 levels correlated with poor prognosis that, however, did not reach significance (Supplementary Figure 4d).

### Milciclib targets MYC and CDK2/cyclin A complexes in Group 3 MB

**Fig. 5:**
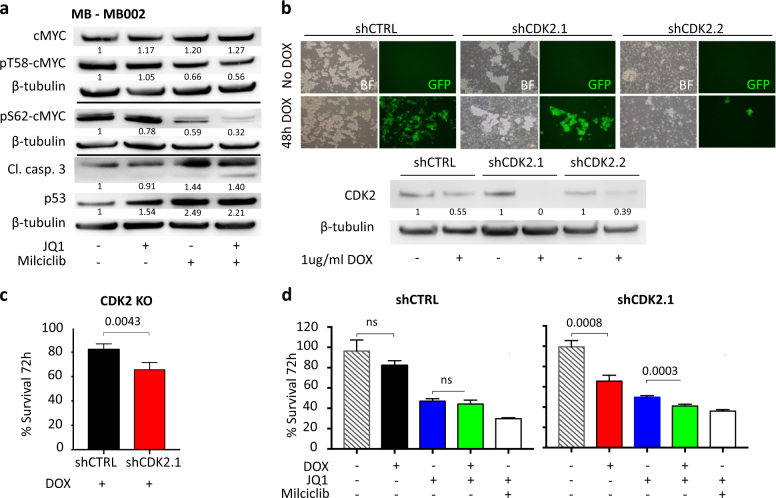
CDK2 inhibition is responsible for dephosphorylation and suppression of MYC in human Group 3 tumor cells. **a** Protein levels of total MYC, p-T58 MYC, p-S62 MYC, p53, and cleaved caspase 3 in 24 h treated MB002 cells. **b** Forty-eight-hour DOX-induction of transduced MB002 with shRNA targeting CDK2 or non-targeting control. Verification of downregulated CDK2 expression using western blot. **c** Survival of MB002 cells after 72 h DOX induction of shRNA CDK2 (shCDK2) or non-targeting shRNA control (shCtrl). **d** Survival of MB002 cells after 72 h DOX induction of shCtrl and shCDK2.1 alone or together with JQ1 (500 nM) or Milciclib (500 nM)

We next wanted to investigate what effect our treatment had on MYC stabilization. Following 24 h of treatment of MB002, levels of pS62-MYC were reduced by JQ1 and Milciclib as compared to controls. Moreover, Milciclib further reduced pT58-MYC levels as compared to control. By using the two compounds in combination, pS62-MYC expression was completely eliminated (Fig. 5a). Total MYC levels were, however, only reduced after 72 h and not after 24 h of treatment (Fig. 5a, Supplementary Figure 5a). There was also a concomitant increase in p53 and the apoptotic marker cleaved caspase-3 in treated MB002 cells compared to control (Fig. 5a). In order to see whether CDK2 alone was responsible for the treatment effects seen when treating MB002 with Milciclib, we targeted CDK2 using DOX-inducible short hairpin RNAs (shRNAs) (Fig. 5b). shRNA targeting of CDK2 significantly reduced MB002 cell survival (Fig. 5c) and a combination of JQ1 and DOX-induced shCDK2 significantly decreased the survival of MB002 as compared to JQ1 alone (Fig. 5d).

Milciclib selectively not only inhibits CDK2/cyclin A at nanomolar levels (IC50 of 45 nM) but also shows efficacy (with IC50 of 53 nM) on Tropomyosin receptor kinase-A (TrkA/Ntrk1) [26]. However, 6 h Milciclib treatment alone or in combination with JQ1 did not reduce the expression levels of Ntrk1 in MB002 cells (Supplementary Figure 5b). Further, treatment with GW441756, a potent inhibitor of TrkA (with IC50 of 2 nM), did not significantly suppress MB002 proliferation after 72 h, not even in micromolar concentrations (Supplementary Figure 5c) [35]. It has been suggested that BET inhibitors also synergize with CDK9 inhibitors and induce apoptosis through a MYC-independent mechanism in killing cells [36]. MB002 and GTML2 cells were sensitive to direct CDK2 targeting and to the drug Dinaciclib (a CDK2/CDK9 inhibitor) at nanomolar levels (Supplementary Figures 5d,e). These tumor cells did, however, not respond to CDK9 inhibition when we used the highly specific CDK9 inhibitor LDC000067, previously reported to target MYC (Supplementary Figures 5d,e) [37].

### Long-term combination therapy abolish the risk of tumor cell recovery

**Fig. 6:**
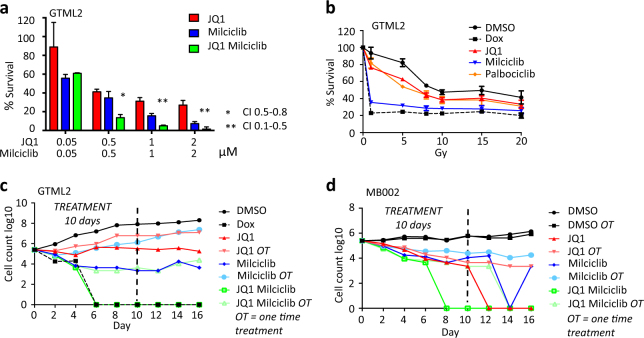
JQ1 and Milciclib treatment synergistically target MYC-driven medulloblastoma cells. **a** Survival of GTML2 cells after treatment with the indicated concentration of JQ1 and Milciclib. Combination index (CI) was calculated using the CompuSyn software for drug combinations and for general dose effect analysis, ComboSyn, Inc. Paramus, NJ, 2007. [www.combosyn.com*]*. *Indicate CI: 0.5–0.8 moderate synergy; **CI: 0.1–0.5 strong synergy. **b** Dose response (survival) of JQ1, Milciclib, or Palbociclib treatment together with single-dose irradiation in GTML2, response compared to non-irradiated DMSO control, analyzed 5 days postirradiation and/or posttreatment. **c** Long-term treatment of GTML2 cells with JQ1, Milciclib, or Palbociclib alone or in combination. **d** Long-term treatment of MB002 cells with JQ1 (500 nM), Milciclib (500 nM), or Palbociclib (2 μM) alone or in combination. **c**, **d** Cells treated one time (OT) or every other day for 10 days and monitored for tumor cell recovery until 16 days posttreatment start

Results from the RNA-Seq analysis show that JQ1 and Milciclib inhibit MYC in rather different ways, suggesting an additive or synergistic effect. Indeed, when using a parallel set of decreasing concentrations of the two inhibitors alone or in combination we saw that JQ1 and Milciclib synergistically reduced GTML2 cell survival (Fig. 6a), whereas the PAN-CDK inhibitor, Purvalanol A, did not act in synergy with JQ1 (Supplementary Figure 6a).

As radiotherapy is an important part of MB standard treatment, it would be important to know how these cells respond to irradiation and if the effect of our MYC-targeted drug treatment is affected by increasing levels of irradiation. The mouse-derived cell lines were indeed sensitive to radiation (0–20 Gy) as shown in the cell cycle analysis and the single-dose response curves (Supplementary Figures 6b,c). GTML2 cells showed an increased response when using irradiation together with single-agent treatment (Fig. 6b) compared to non-irradiated DMSO control. However, DOX and Milciclib treatment in combination with irradiation showed the lowest additive response when compared to its non-irradiated treatment equivalent in GTML2 (Supplementary Figure 6d). A 20 Gy dose only improved DOX or Milciclib treatment with 8.3 and 35%, respectively, compared to 70.3% improvement of Palbociclib treatment in GTML2 cells.

A successful cancer treatment leaves no room for survival of dormant tumor clones that will cause relapse or treatment resistance [38]. We therefore studied the effects of JQ1 and Milciclib or Palbociclib treatment alone or in combination for a longer time under controlled culture conditions. GTML2 and MB002 cells were cultured, serially passaged, and simultaneously treated over 16 days in vitro (Fig. 6c, d; Supplementary Figures 6e,f). Continuous single-agent treatment alone could not abolish tumor cell recovery in GTML2 (Fig. 6c). By contrast, continuous combination treatment eliminated viable tumor cells after 6 days when using JQ1 and Milciclib and after 9 days when using JQ1 and Palbociclib. GTML2 cells never recovered, showing how continuous JQ1 and Milciclib or Palbocicilb combination treatment worked as efficiently as DOX treatment. Similarly, long-term treatment of human MB002 cells showed continuous combination treatment to be the most effective treatment, abolishing tumor cell recovery after 8 days of treatment with JQ1 and Milciclib or Palbociclib (Fig. 6d, Supplementary Figure 6f).

### Combination treatment significantly prolong survival in human Group 3 MB

**Fig. 7:**
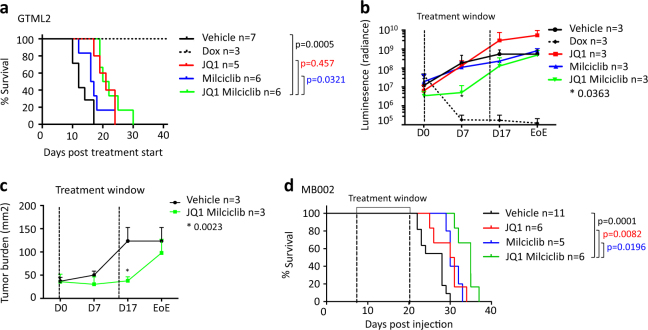
Dual BET and CDK2 inhibition of orthotopically grafted Group 3 tumors leads to a significantly prolonged survival. **a** Kaplan–meier survival curve of GTML2 xenografts treated with 7 doses of JQ1 or Milciclib or a combination of the two. **b** Bioluminescence measurement (luminescence) of luciferase in GTML2-transplanted mice from the start of treatment until the end of experiment (EoE). **c** Tumor growth was monitored using luciferase expression in transplanted cells during the course of treatment of GTML2 xenografts. The tumor area (mm^2^) was calculated based on luciferase signal from the NightOWL IVIS (Berthold) and analyzed using the Indigo software. **d** Kaplan–Meier survival curve of MB002 xenografts treated with seven doses of JQ1, Milciclib, or a combination of both compounds. Kaplan–meier curve statistical analysis: LogRank (Mantel–Cox test); luminescence and tumor burden statistical analysis: Student’s *t*-test

As both JQ1 and Milciclib were able to penetrate the blood–brain barrier (BBB) [10, 31], we were further interested to see whether the drugs were able to show efficacy on MYCN- or MYC-driven tumors growing in the brain. Mice were orthotopically injected into the cerebellum with 100,000 luciferase- and MYCN-positive, stable GTML2 tumor cells or human MYC-amplified MB002 cells as previously described [13, 14]. Combination treatment significantly prolonged survival in GTML2 allografted mice (Fig. 7a) compared to vehicle controls. Interestingly, bioluminescent analysis of GTML2 tumors treated with combination therapy showed an initial reduction in luminescence intensity and tumor size during the time of treatment (Fig. 7b, c, Supplementary Figure 7a); however, posttreatment, the tumor burden levels recovered to vehicle levels. Moreover, equimolar concentrations of JQ1/Milciclib and JQ1/Cisplatin reduce cell survival of MB002 to the same extent in vitro (Supplementary Figure 7b). Cisplatin treatment of MB002 xenografts did not increase survival in mice compared to vehicle control (Supplementary Figure 7c). However, as few as 7 doses of JQ1 and/or Milciclib significantly prolonged survival in MB002 compared to vehicle (Fig. 7d). Interestingly, combination therapy, using JQ1 together with Milciclib, further increased survival as compared to single-agent therapy, which suggested that the drugs worked in synergy in order to suppress tumor growth also in vivo.

## Discussion

MYC proteins are considered un-druggable and lack obvious pockets where small molecules or drugs can bind [7]. MYC proteins are transcription factors with very short half-lives that are rapidly targeted by specific degradation by the ubiquitin proteasome system [39]. With these caveats in mind, interruption of MYC-dependent pathways and *MYC* regulatory units might be a promising alternative to indirectly target MYC proteins in cancer.

The BET family consists of four different bromodomain-containing proteins, which are important in several cellular processes such as mitosis and transcriptional regulation [40]. JQ1 exerts its inhibitory effect by displacing the BET bromodomains from the chromatin through competitive binding to the acetyl-lysine recognition pocket [41]. BET inhibition caused by JQ1 results in downregulation of *MYC* transcription after 24 h [10] leading to downregulation of MYC target genes in MB cells. However, 24 h is a rather long time point for studying direct effects of gene regulation. In the search for direct targets and transcriptional regulators in our MB models, we found that JQ1 could not downregulate MYC or MYCN itself after a shorter 6 h treatment. However, JQ1 targeted the output of MYC/MYCN transcription in a similar way as when MYCN was depleted by using 6 h DOX regulation. JQ1 could still inhibit MYC or MYCN levels after 24 or 72 h in both genetically engineered GTML2 tumor cells and in MYC-amplified MB002 cells.

CDKs regulate events in MYC function, MYC processing, and are key players in cell cycle progression [42]. Interestingly, recent reports have shown good efficacy of using specific CDK inhibition in MYC-amplified Group 3 MB. For example, the CDK4/6 inhibitor, Palbociclib, was recently shown to efficiently target MYC in grafted serum-cultured classical MYC-amplified cell lines D283 and D425 or in MYC-transformed NSCs. [43]. Our data suggest that not only Palbociclib but also the CDK2-specific inhibitor Milciclib is efficiently inducing apoptosis in tumor lines cultured in serum-free conditions. In our MB models, MYC and MYCN genes themselves were not suppressed transcriptionally by the Milciclib treatment; however, MYC target genes were downregulated presumably from destabilization of MYC/MYCN proteins caused by suppressed phosphorylation of MYC at residue S62 following CDK2 inhibition as previously reported [16]. We saw that the inhibitory effect was mimicked by suppressing CDK2 by using specific shRNAs and further found that neither TrkA nor CDK9 was involved in the mechanisms of tumor cell suppression.

Our results suggest a combined treatment approach in order to efficiently target MYC-dependent pathways preferably in MYC- or MYCN-driven Group 3 and Group 4 MB where these pathways are active. Both JQ1 and Milciclib passed the BBB (as previously reported [10, 31]), were well tolerated, reduced tumor cell growth, and significantly prolonged survival in animals. BET inhibitors similar to JQ1 such as RG6146 (aka. TEN-010) or OTX105 are currently in clinical trials [ClinicalTrials.gov NCT01987362, NCT02259114]. Further, Milciclib is/has been used in clinical trials [NCT01011439, NCT01301391] and report considerably moderate and reversible side effects from the treatment [44]. As presented in this study, JQ1 and Milciclib suppress MYC in different ways, causing a synergistic inhibition rather than an additive repression. We therefore propose using these inhibitors in combination for treating MYC-dependent, aggressive pediatric brain tumors.

## Materials and methods

### Cell lines

MYCN-driven mouse MB cells and hindbrain NSCs were derived and cultured as previously described [14]. DAOY and D283 were cultured in Dulbecco’s modified Eagle’s medium supplemented with 10% serum and PeSt. Human hindbrain NSCs, Sai2, and human induced pluripotent stem-derived cells, AF22, were provided by Dr. Anna Falk (Karolinska Institutet, Sweden) and was cultured as previously described [45]. MB002 cells were obtained from Dr. Cho, Stanford and cultured as previously described [10]. Further, CHLA259 was obtained from Children’s Oncology Group Cell Culture and Xenograft Repository, Texas, USA; Kelly neuroblastoma cells obtained from ATCC (Wesel, Germany); and human cerebellar astrocytes (HA-c) and human spinal cord astrocytes (HA-sp) were acquired from Sciencell Research Laboratories, Carlsbad, CA.

### Transcriptome analysis

MB cells were treated 2 h with DMSO or underwent 6 h treatment with DMSO, JQ1 (500 nM), Milciclib (500 nM), both aforementioned compounds in combination, or DOX (1 μg/ml). RNA was purified using the RNeasy Kit (Qiagen). RNA sequencing was performed using the Ion Proton™ System for Next-Generation Sequencing and run at NGI, Science for Life Laboratory, Uppsala Biomedicinska Centrum (BMC), Sweden. All RNA sequence reads were processed as previously described [46]. All treatment conditions were submitted and processed in triplicates. However, after quality controls, one replicate of DOX-treated cells was removed due to inferior quality. GEO accession number: GSE107405.

### Differential expression analysis

The differential expression results obtained from Cuffdiff were processed using the R package CummeRbund [47]. Fold changes (FCs) for each comparison were recalculated using expression values equal to FPKM + 1 to avoid results including infinity. For improved stringency on selection, we employed an expression cutoff corresponding to the median expression across all GTML2 or MB002 samples. The genes with expression above threshold, which was 0.50 and 0.60 in log10(FPKM + 1) units for GTML2 and MB002, respectively, were considered expressed and contained on average 50% of all genes [48]. Transcripts were considered significantly differentially expressed, if the recalculated FC > 1.5 or FC > 1.25 for GTML2 and MB002, respectively, if the FDR-adjusted *p*-value *q* < 0.05, and if the expression (FPKM + 1) in at least of the two conditions was above the respective threshold.

### Mapping of orthologs and translation of human gene symbols

Mapping of mouse genes to human orthologs and human genes to their official gene symbols was performed as previously described [46].

### Gene set enrichment and GSO analyses

GSEA and GSO analyses were performed as previously described [46]. Unless otherwise specified, unbiased GSEA and GSO were performed against four different databases of GSEA gene sets: H (hallmark gene sets), C2 (curated gene sets), TFT (transcription factor targets), and C6 (Oncogenic signatures). To test the regulation of MYCN target genes, four MYCN-related gene sets were selected (COWLING_MYCN_TARGETS, KIM_MYCN_AMPLIFICATION_TARGETS_UP, NMYC_01, WEI_MYCN_TARGETS_WITH_E_BOX) and used for targeted GSEA. To perform GSEA against MB Group 3- and Group 4-related gene sets, MB signature genes were downloaded [49], and the 50 top ranking genes for Group 3 and Group 4 were selected as Group 3 and Group 4 gene sets, respectively.

For GSEA performed against individual target gene sets, an enrichment with a *p*-value *p* < 0.05 was considered significant. In unbiased GSEA and GSO screens, an enrichment with the FDR-corrected *p*-value FDR < 0.05 was considered significant.

### Viability assay

Cell viability was measured using 1:10 Resazurin reagent; fluorescence was detected by excitation at 530 nm and emission at 590 nm. Inhibitors/concentrations used: JQ1 (500 nM) (Bradner, Harvard Medical School), Milciclib (PHA848125) (500 nM) (Nerviano Medical Sciences), Palbociclib (PD0332991) (2 µM) (ActiveBiochem), Purvalanol A (10 µM), Dinaciclib (200 nM) (Selleckchem), LDC000067 (500 nM) (Selleckchem), and DOX (1 µg/ml) (Sigma). Concentration curves: JQ1 (0–500 nM), Milciclib (0–15 μM), Palbociclib (0–15 μM), and DOX (0.01–1000 ng/ml) analyzed 72 h posttreatment. Data were analyzed using the GraphPad Prism6 software using Students’ *t*-test.

### Irradiation

Cells were irradiated using ^137^Cs ɣ-radiation (Gammacell® 40 Exacor) at dosage 1 Gy/min. Irradiation, 0–20 Gy, was administered either alone or in combination with JQ1 (500 nM), Milcicib (2 µM), and Palbociclib (2 µM). After irradiation and addition of inhibitors, cells were incubated for 5 days before viability or cell cycle analysis.

### Long-term treatment

Two hundred thousand cells were seeded with inhibitors JQ1 (500 nM), Milciclib (500 nM), and Palbociclib (2 µM) either alone or in combination. Cells were dissociated and counted every 48 h and then resuspended in fresh medium containing inhibitor/s or only in new media (OT-groups). Inhibitors were added for 10 days after which cells were monitored for 6 additional days.

### Western blot

Twenty micrograms of protein was loaded in 4–12% Bis-Tris gels (NuPAGE) and transferred to iBlot nitrocellulose filter (Invitrogen). Primary antibodies: Cyclin A (ab38, 1:500), MYCN (ab16898, 1:250), c-MYC (sc-764, 1:1000), pS62-MYC (ab51156, 1:500), pT58-MYC (ab28842, 1:500), CDK2 (05–596, 1:500, Millipore), p53 (sc-126, 1:1000), cleaved caspase 3 (9661S, 1:500, CST), β-Actin (sc47778, 1:1000), and β-Tubulin (MAB3408, 1:500 and 2146, 1:1000, CST). ECL secondary antibodies (1:5000) (GE healthcare) were detected using Supersignal West Pico Chemiluminescent substrate (ThermoFisher Scientific). Quantification was performed using ImageJ by using the ratio of the sample relative density and the loading control relative density.

### Intracellular staining and cell cycle analysis

Inhibitors were added as single treatment or as combinations of JQ1 (500 nM), Milciclib (500 nM), and Palbociclib (2 μM) with DMSO control to cell culture plates. Cells were stained with propidium iodide for cell cycle analysis and for intracellular staining with Ki67 (ab16667, 1:100), Luciferase (L2164, 1:100, Sigma), and cleaved caspase-3 (9661S, 1:100, CST). Cells were fixed and permeabilized using FIX&PERM (Invitrogen) after 72 h treatment. Alexa488 and Alexa555 (1:2000) secondary antibodies were used for detection. Fifty thousand events per treatment was recorded using BD LSR II multi-laser flow cytometer (BD Biosciences).

### Inducible lentiviral shRNA

Lentiviral SMARTchoice Inducible Human CDK2 shRNA, shCDK2.1 (sh67127 gene target seq. GCCAGAAACAAGTTGACGG), shCDK2.2 (sh68543 gene target seq. ACACGTTAGATTTGCCGTA), or a SMARTvector Inducible Non-targeting control (VSC6570) (Dharmacon, GE Life Sciences) was used to transduce MB002 cells. Transduced cells were under puromycin selection for 10 days. Cells were DOX-induced (1 μg/ml) at time 0 (where applicable), and viability was measured using resazurin at the indicated times.

### In vivo MB xenografts

In vivo studies were performed in accordance with approved protocols from the Regional Ethical Review Board in Uppsala, Sweden. Briefly, 100,000 MB002 or MB-GTML cells were injected into cerebella (as described in ref. [14]) of 6–8-week-old female Athymic Nude-Foxn1nu mice (Harlan Laboratories). Seven days postinjection, mice were randomized into groups and administered JQ1 (50 mg/kg), Milciclib (10 mg/kg), JQ1 together with Milciclib (50 mg/kg and 10 mg/kg, respectively) or vehicle alone (DMSO) in 10% HP-β-Cyclodextrin (Sigma) on alternating days via intraperitoneal injection for 14 days. Cisplatin 2 mg/kg (Sigma) in DMSO:10% HP-β-Cyclodextrin was administered weekly four times. Statistical analysis of Kaplan–Meier survival curves was performed using the log-rank (Mantel–Cox) test.

## Electronic supplementary material

Supplementary Figure 1

Supplementary Figure 2

Supplementary Figure 3

Supplementary Figure 4

Supplementary Figure 5

Supplementary Figure 6

Supplementary Figure 7

Supplementary table 1

Supplementary table 2a

Supplementary table 2b

Supplementary figure legends and material and methods
