# Supplementary figures and images for: Combined BET bromodomain and CDK2 inhibition in MYC-driven medulloblastoma
Source: Oncogene. 2018 Mar 7;37(21):2850–62. doi: 10.1038/s41388-018-0135-1 (PMC5966365; doi:10.1038/s41388-018-0135-1)

# Supplementary Figure 1

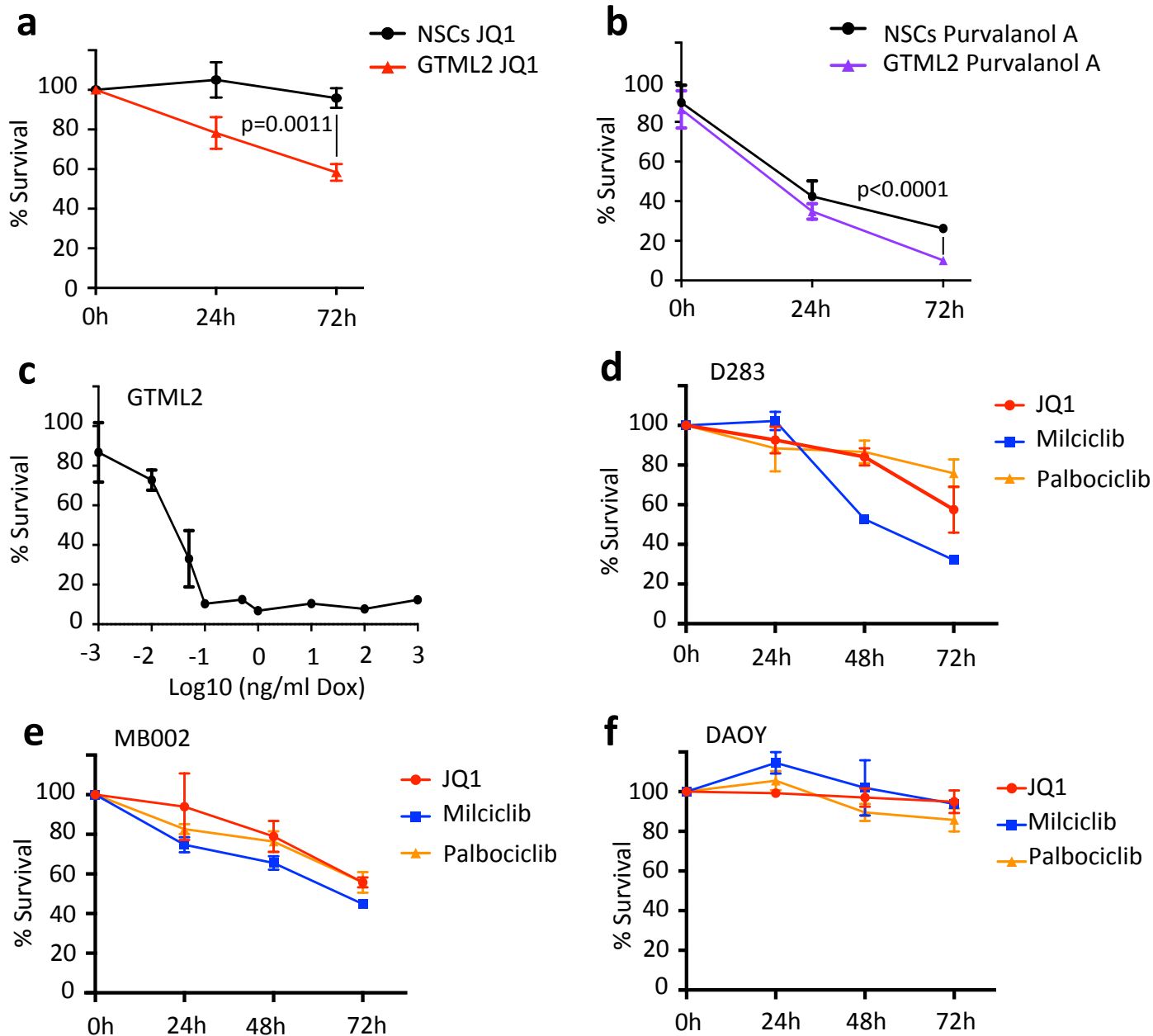

Supplement: Supplementary file 1 — Supplementary Figure 1 [file 41388_2018_135_MOESM1_ESM.pdf]

Supplementary Figure 2

a

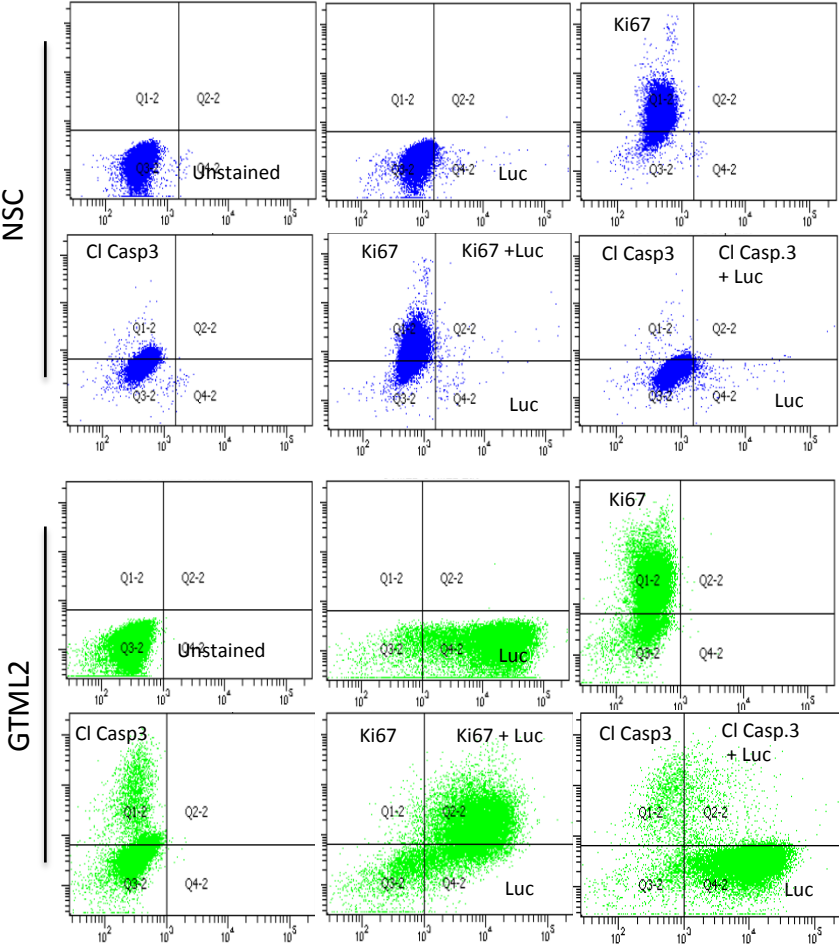

b

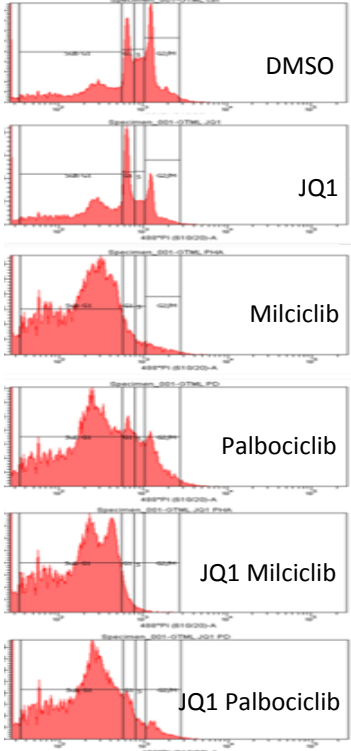

c

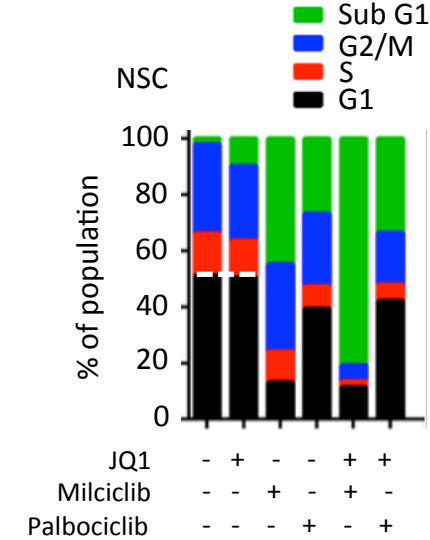

d

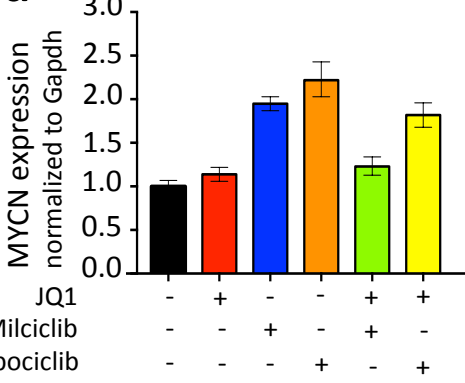

Supplement: Supplementary file 2 — Supplementary Figure 2 [file 41388_2018_135_MOESM2_ESM.pdf]

# a

## Supplementary Figure 3

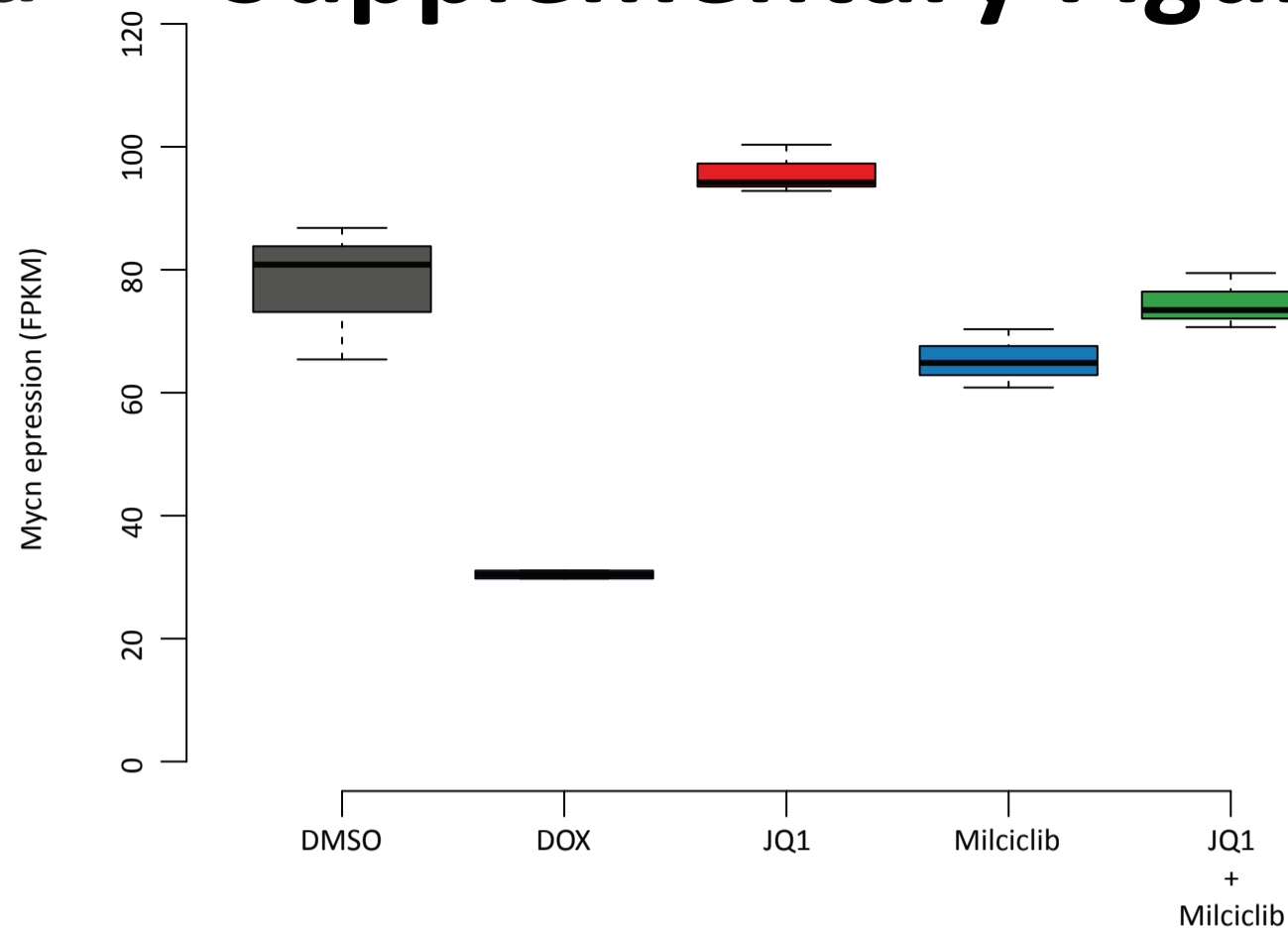

# c

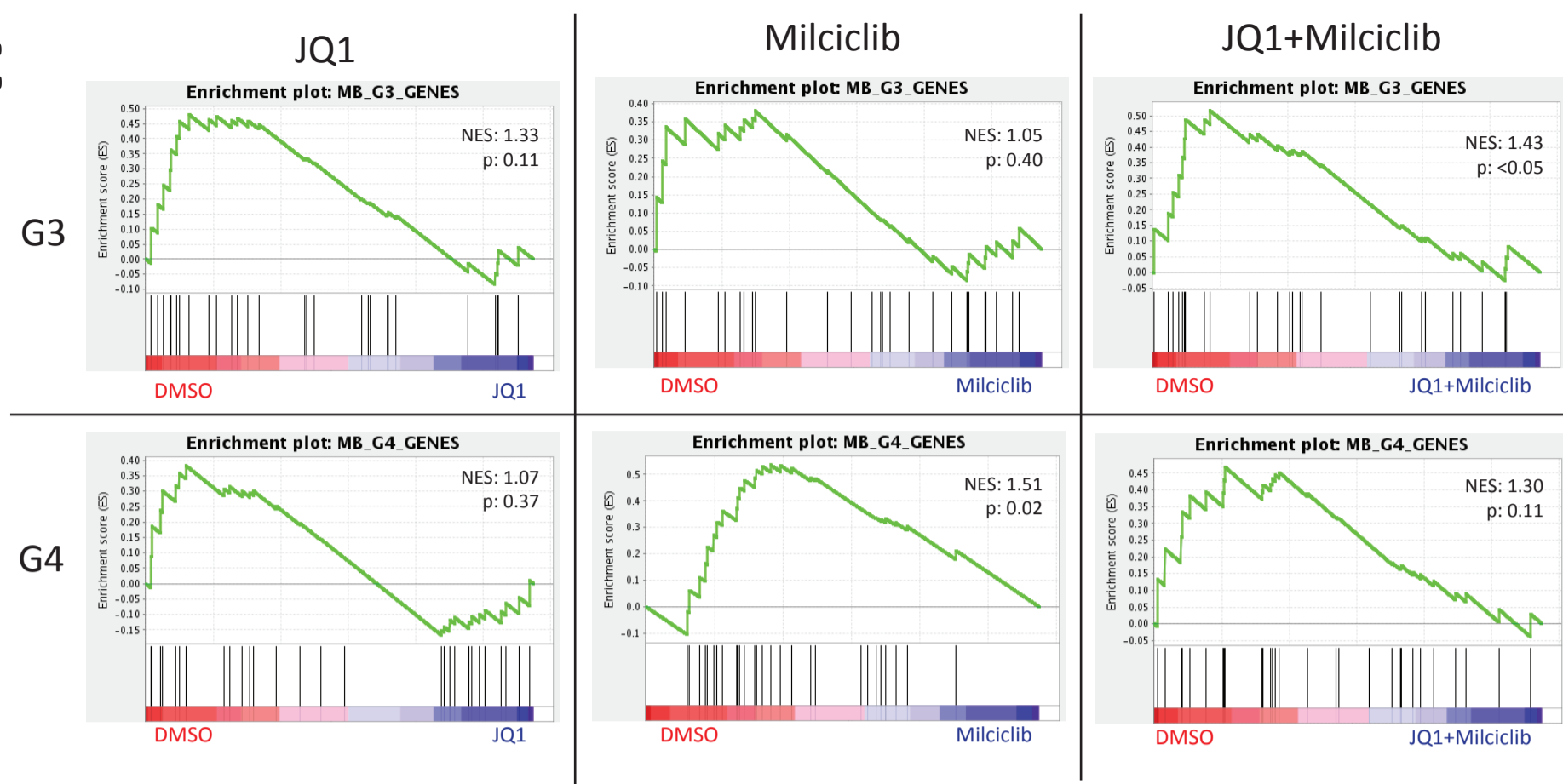

# b

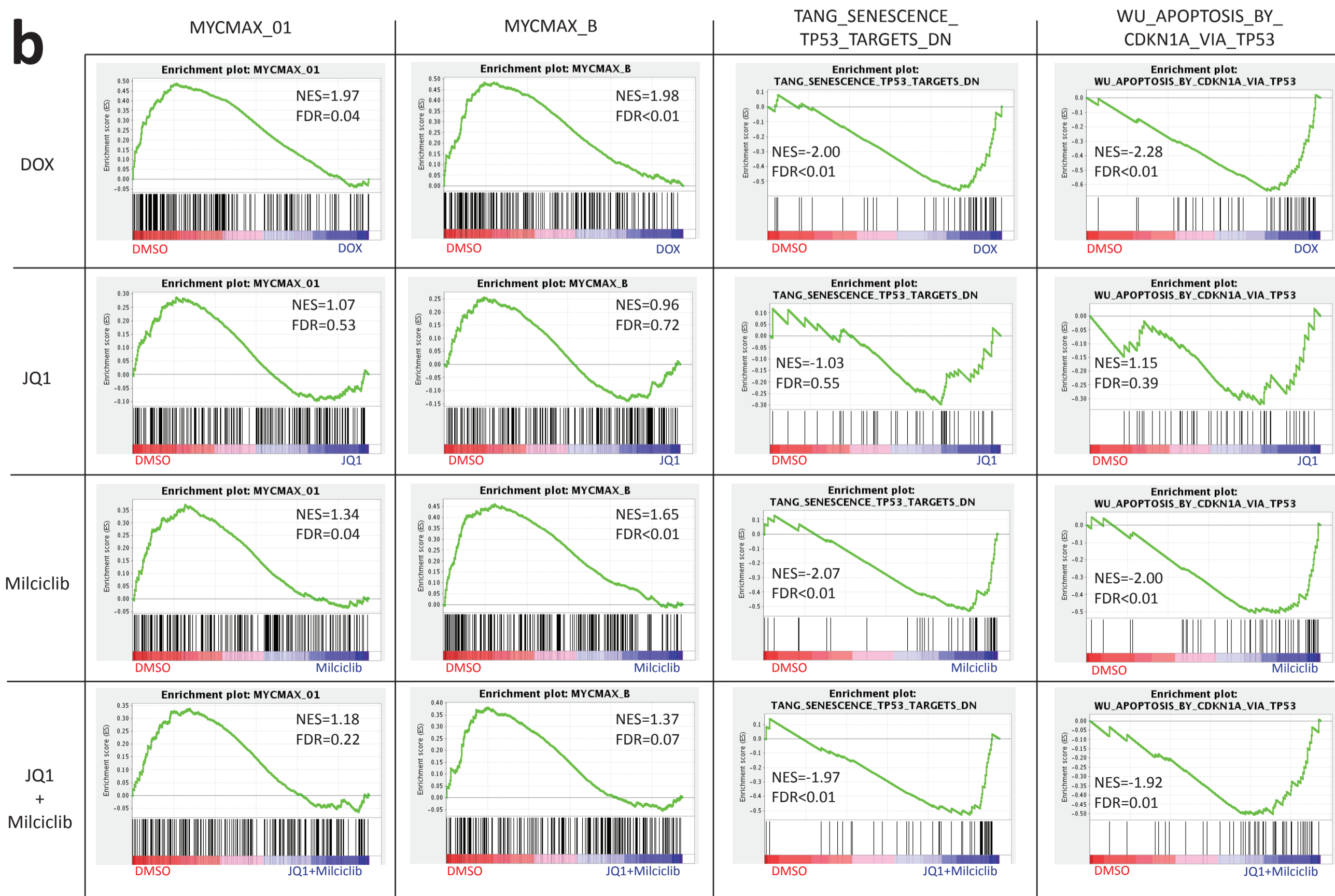

Supplement: Supplementary file 3 — Supplementary Figure 3 [file 41388_2018_135_MOESM3_ESM.pdf]

# a Supplementary Figure 4

**b**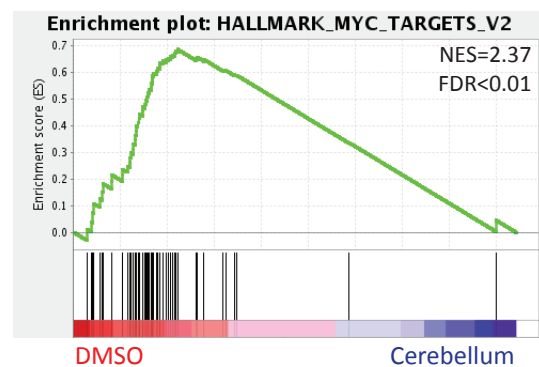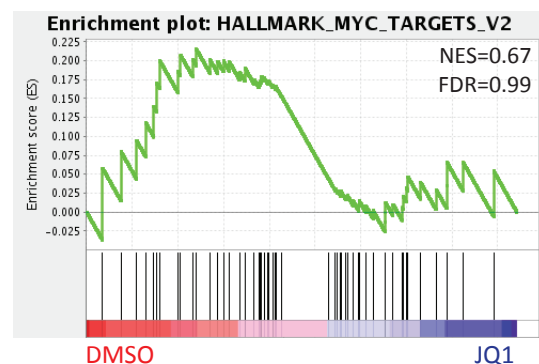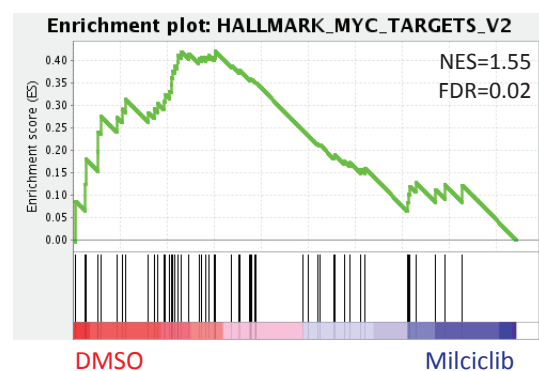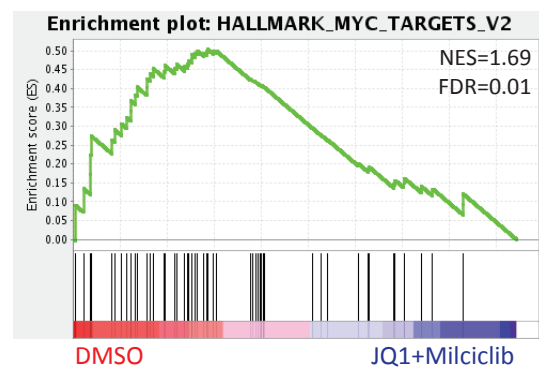

JQ1

G3

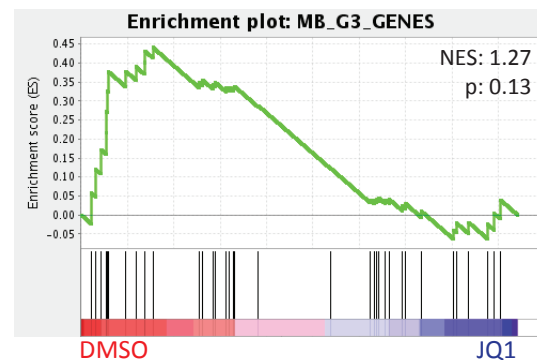

G4

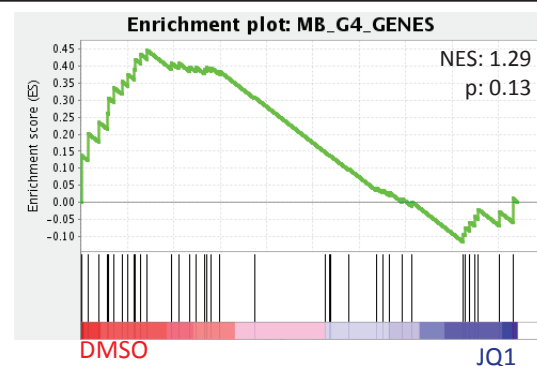

PHA

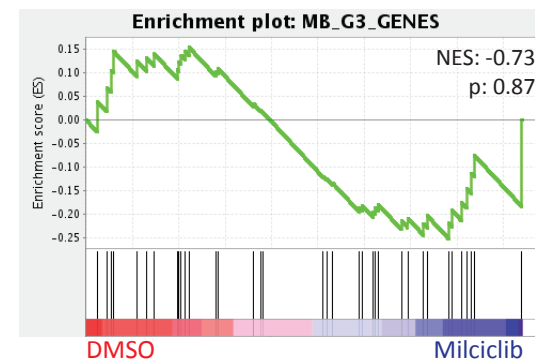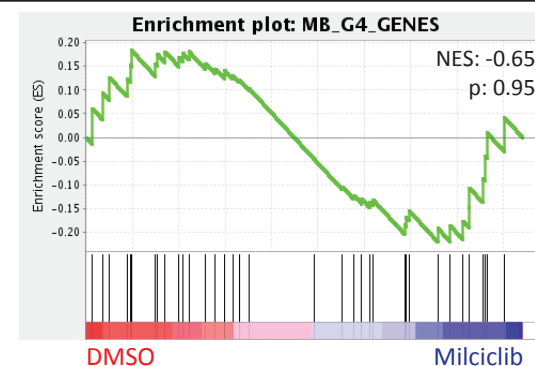

JQ1PHA

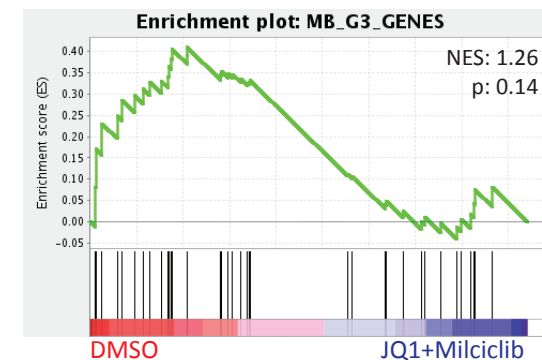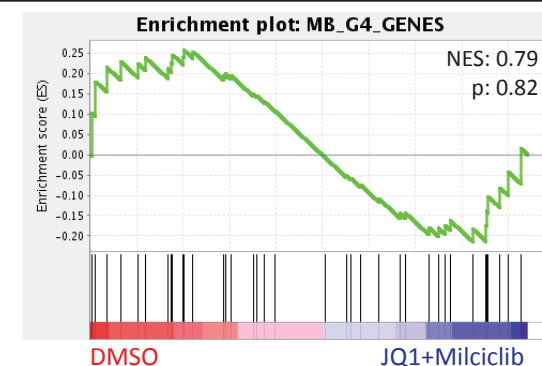**c**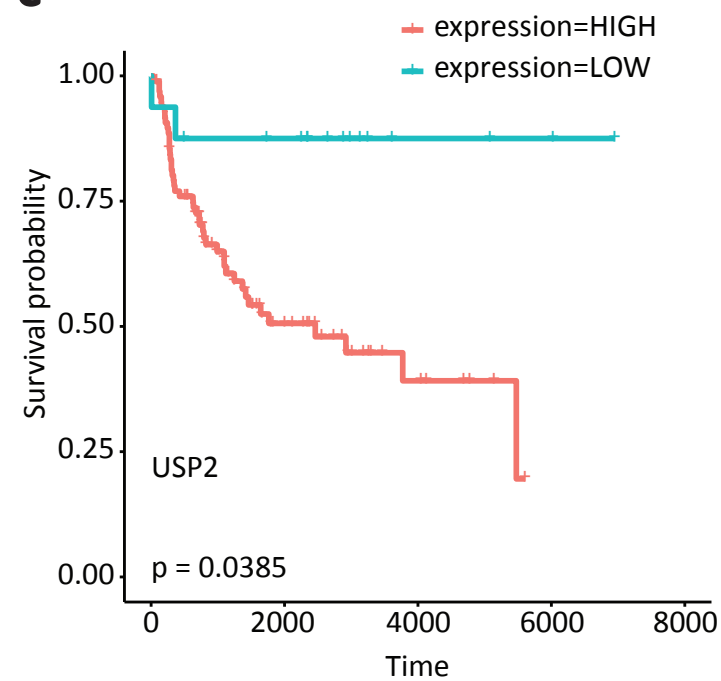**d**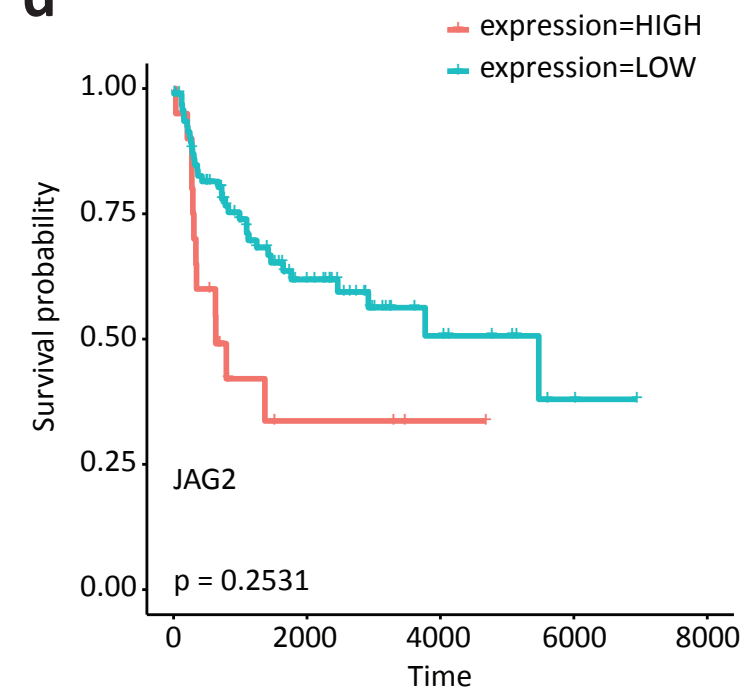

Supplement: Supplementary file 4 — Supplementary Figure 4 [file 41388_2018_135_MOESM4_ESM.pdf]

# Supplementary Figure 5

**a**

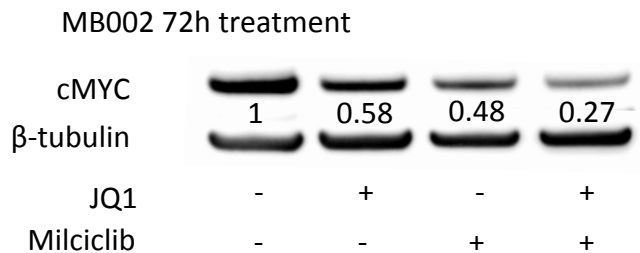

**b**

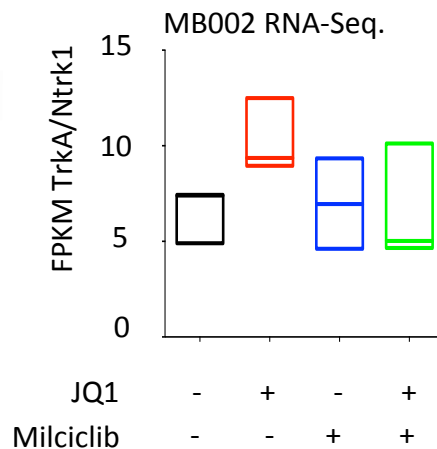

**c**

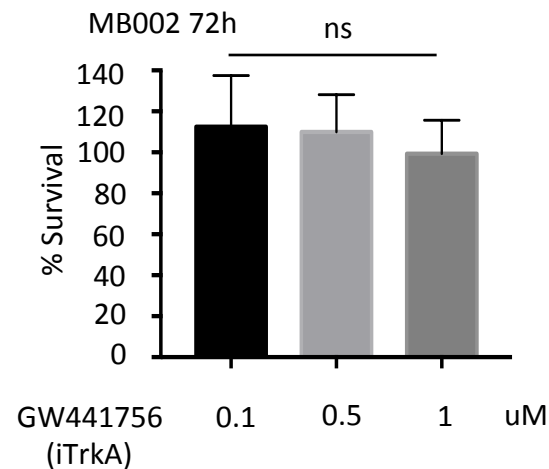

**d**

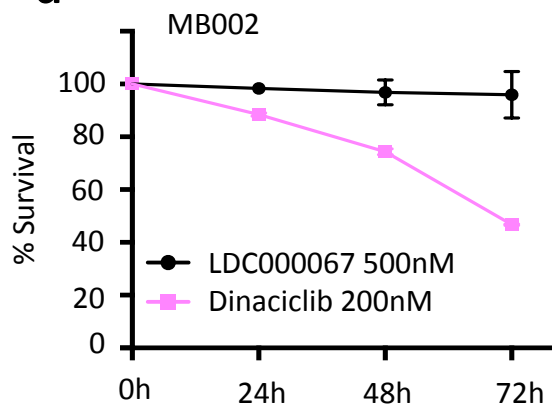

**e**

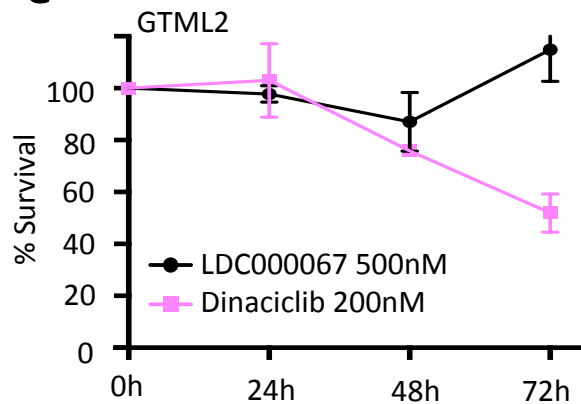

Supplement: Supplementary file 5 — Supplementary Figure 5 [file 41388_2018_135_MOESM5_ESM.pdf]

# Supplementary Figure 6

**a**

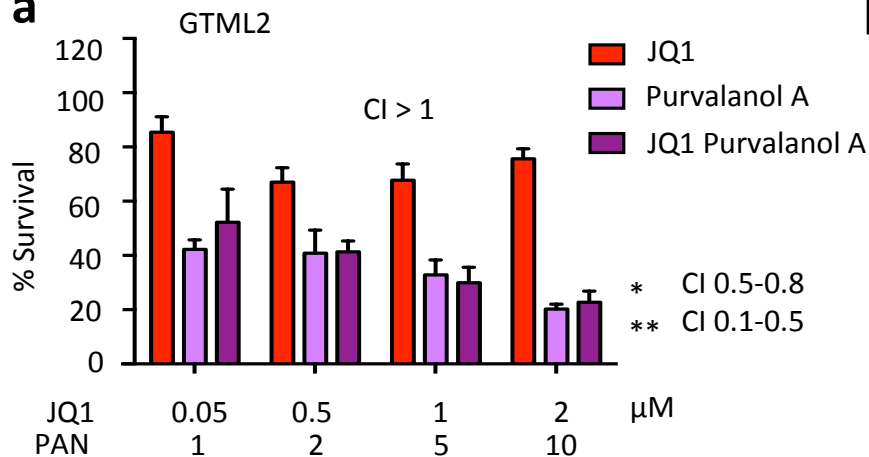

**b**

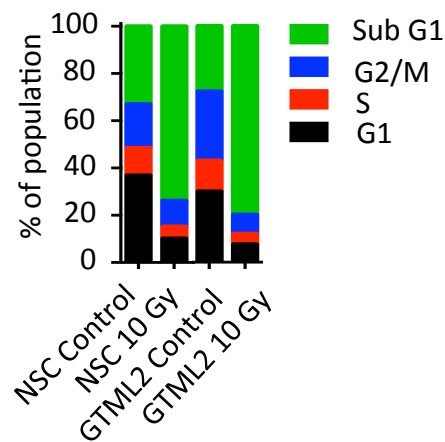

**c**

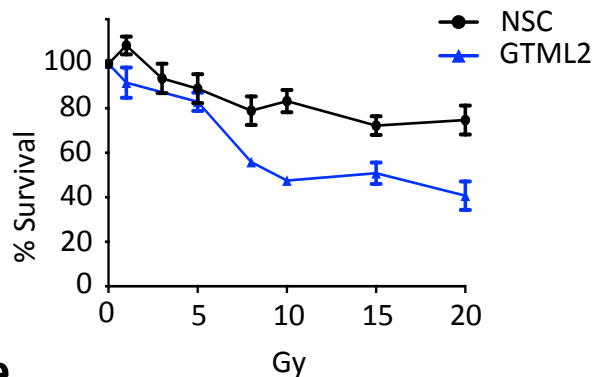

**d**

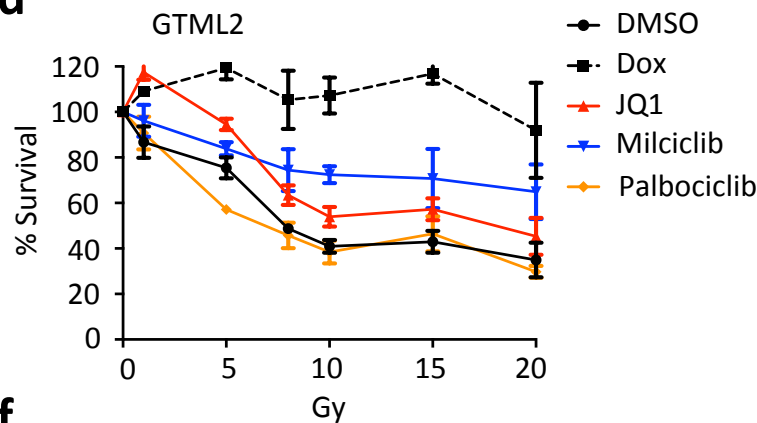

**e**

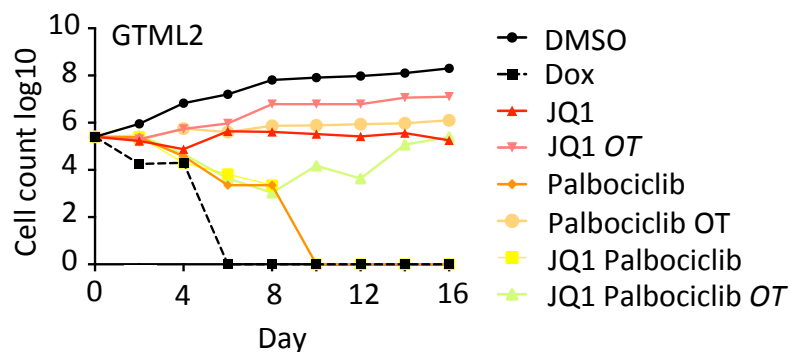

**f**

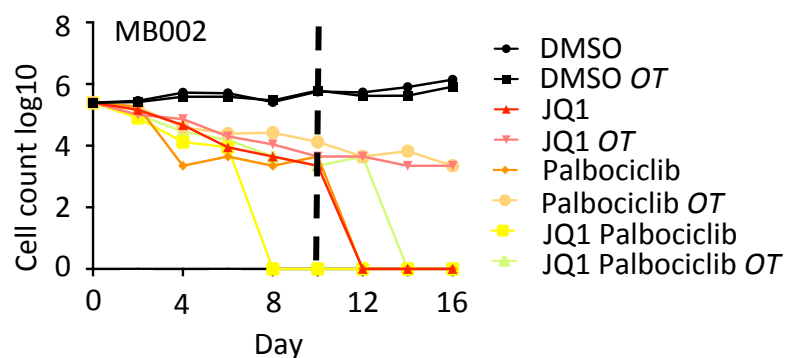

Supplement: Supplementary file 6 — Supplementary Figure 6 [file 41388_2018_135_MOESM6_ESM.pdf]

## Supplementary Figure 7

**a**

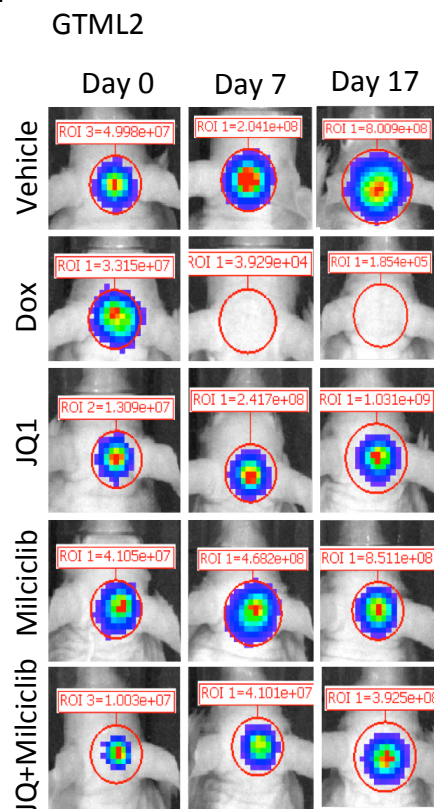

**b**

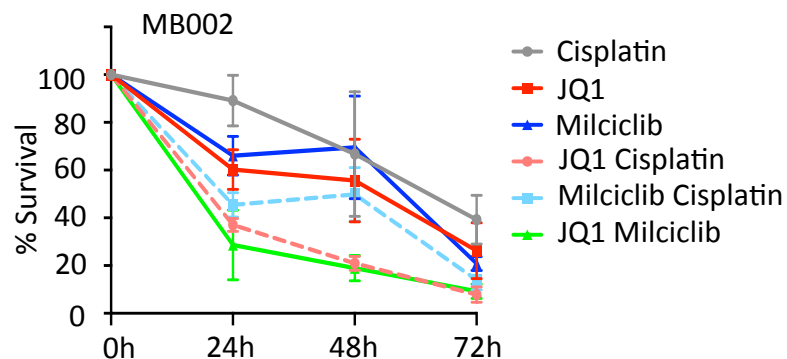

**c**

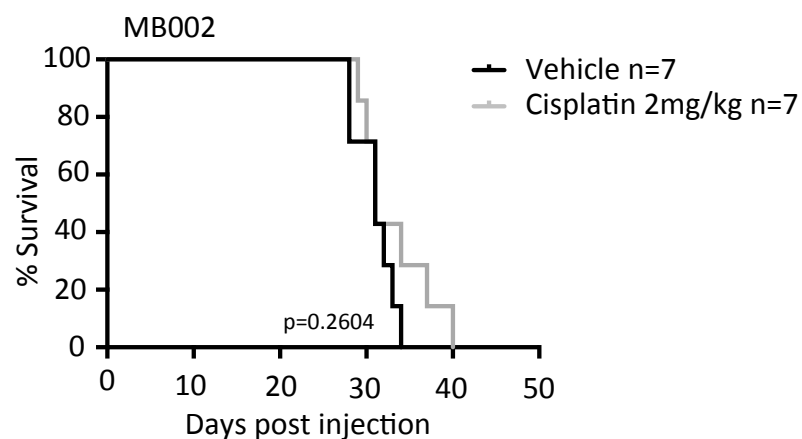

Supplement: Supplementary file 7 — Supplementary Figure 7 [file 41388_2018_135_MOESM7_ESM.pdf]
