# Supplementary material for: Combined BET bromodomain and CDK2 inhibition in MYC-driven medulloblastoma
Source: Oncogene. 2018 Mar 7;37(21):2850–62. doi: 10.1038/s41388-018-0135-1 (PMC5966365; doi:10.1038/s41388-018-0135-1)
Supplement: Supplementary file 11 — Supplementary figure legends and material and methods [file 41388_2018_135_MOESM11_ESM.docx]

**Supplementary data**

**Supplementary Figure legends:**

**Supplementary Figure 1.** Response of normal NSC and GTML2 to **a.** JQ1 and **b.** PAN CDK inhibitor, Purvalanol A. **c.** Dose-response curves of GTML2 treated (Tet-OFF system) with DOX for 72h. Survival of human **d.** D283, **e**. MB002, **f**. DAOY with JQ1, Milciclib or Palbociclib treatment.

**Supplementary Figure 2.** **a.** Intracellular staining of normal NSC and GTML2 identifying populations positive for luciferase, Ki67 and cleaved caspase-3. Detected using Alexa488 (x-axis) and Alexa555 (y-axis) secondary antibodies. **b.** Cell cycle histogram showing gating of GTML2 cell populations; quantified in Figure 2. **c.** Cell cycle analysis of normal NSC after 72h of single or combination treatments with JQ1, Milciclib and Palbociclib. **d.** Quantification of MYCN mRNA expression in GTML2 following 72h treatment with JQ1, Milciclib and Palbociclib; expression normalized to Gapdh.

**Supplementary Figure 3. a.** MYCN expression of GTML2 cells treated 6h with DMSO, DOX, JQ1, Milciclib or JQ1+Milciclib. **b**. GSEA results for two TP53/apoptosis gene sets and two MYC target genes related gene sets, which were found significantly upregulated or downregulated, respectively in GTML-Dox and GTML-Milciclib but not GTML-JQ1 as compared to GTML-DMSO; gene sets were identified from an unbiased GSEA analysis against four gene set databases (Supplementary table 1). Enrichments were considered significant if FDR<0.05.  **c.** GSEA results comparing GTML-DMSO against GTML-JQ1, GTML-Milciclib, or GTML-JQ1+Milciclib on a gene set of MB group 3 signature genes (top panel) or MB group 4 signature genes (bottom panel). Enrichments were considered significant if p<0.05

**Supplementary Figure 4**. **a.** GSEA for a MYC target genes related gene set comparing MB002-DMSO against cerebellar cells, MB002-JQ1, MB002-Milciclib, or MB002-JQ1+Milciclib; the gene set was identified from an unbiased GSEA on four different gene set databases (Supplementary Table 4a) as significantly upregulated in both cerebellar cells and MB002-JQ1+Milciclib as compared to MB002-DMSO+Milciclib. Enrichments were considered significant if FDR<0.05. **b.** GSEA results comparing MB002-DMSO-6h against MB002-JQ1-6h, MB002-Milciclib-6h, or MB002-JQ1+Milciclib on a gene set of MB group 3 signature genes (top panel) or MB group 4 signature genes (bottom panel). Enrichments were considered significant if p<0.05. In a cohort of tumors from 113 patients ([34](#_ENREF_34)) **c.** USP2 significantly correlated with poor survival in Group 3 MB patients when analyzing patients with high as compared to low USP2 mRNA levels. A similar trend was seen for **d.** JAG2 in where elevated JAG2 levels correlated with poor prognosis that however did not reach significance.

**Supplementary Figure 5.** **a.** Protein expression analysis of MB002 cells treated with JQ1 or/and Milciclib. **b.** Boxplot showing FPKM values of TrkA/Ntrk1 from RNA-Seq in MB002 control and treatment groups. **c.** Survival of MB002 treated with TrkA inhibitor GW441756; statistics - students’ *t*-test. Survival curve of **d.** MB002 and **e.** GTML2 treated with selective-CDK9 inhibitor LDC000067 or PAN CDK inhibitor, Dinaciclib.

**Supplementary Figure 6. a.** Survival of GTML2 cells after treatment with indicated concentration of JQ1 and Purvalanol. Combination index (CI) was calculated using the *CompuSyn software for drug combinations and for general dose effect analysis, ComboSyn, Inc. Paramus, NJ 2007. [www.combosyn.com].* * Indicate CI: 0.5-0.8 moderate synergy; ** CI: 0.1-0.5 strong synergy; CI > 1: no synergy. **b.** Showing the effect of single-dose irradiation on drug treatment efficiency in GTML2. Each treatment is compared to its non-irradiated treatment equivalent to show the contribution of irradiation to the response in cell survival. Survival was analyzed 5 days post irradiation and/or treatment. **c.** Cell cycle analysis of normal NSC, and GTML2 96h after irradiation (10 Gy), compared to non-irradiated controls. **d.** Radiation dose-response curve of normal NSC and GTML2 96h post irradiation. Combined long-term treatment of **e**. GTML2 and **f**. MB002 tumor cells with JQ1 together with selective CDK4/6 inhibitor Palbociclib; treated one time (OT) or every other day for 10 days to follow tumor cell recovery.

**Supplementary Figure 7. a.** Effect of cisplatin on human MB002 together with 500 nM equimolar JQ1 and cisplatin; and 500 nM equimolar Milciclib and cisplatin compared to combination treatment with JQ1 and Milciclib. **b.** Bioluminescent images of treatment effect on MB-GTML tumors, showing DOX to effectively cure these MYCN-dependent tumors. **c.** Kaplan-Meier survival curve of cisplatin-treated MB002 xenografts. Mice were treated with a total of four doses of 2 mg/kg cisplatin, administered i.p once a week. Kaplan-meier curve statistical analysis: LogRank (Mantel-Cox test).

**Supplementary Table 1.** Results of the unbiased GSEA comparing GTML-DMSO-6h against GTML-JQ1-6h, GTML-Milciclib-6h, or GTML-JQ1+Milciclib on four different gene set databases.

**Supplementary Table 2.** **a**. GSEA results comparing MB002-DMSO-6h against MB002-JQ1-6h, MB002-Milciclib-6h, or MB002-JQ1+Milciclib on four different gene set databases. **b.** List of the 71 overlapping genes from figure 4I, which were significantly upregulated in GTML-Dox-6h, GTML-JQ1+Milciclib, and MB002-JQ1+Milciclib as compared to the respective DMSO treatments.

**Supplementary methods:**

**MB subgroup classification**

Projection of RNA-Seq expression profiles onto the four molecular subgroups of MB (WNT, SHH, G3, G4) was conducted using the metagene code for cross-platform, cross-species projection of transcription profiles ([48](#_ENREF_48)) as previously described ([45](#_ENREF_45)). Subgroup classifications of the mouse and human samples were performed separately and their affiliation was determined by the Support Vector Machine (SVM) tool of the metagene software.

**Patient Survival Analyses**

Survival analyses were conducted in R using the survival package. Expression data and survival statistics for MB G3 patients (n=113) were obtained from the GSE85217 dataset. For each gene, the survival between patients with high gene expression and patients with low gene expression was investigated. Specifically, a gene expression cutoff, separating high and low expression, and the corresponding p-value of the resulting survival difference were estimated in R using the maxstat package. Survival differences were considered significant if p<0.05.

**qRT-PCR**

GTML2 cells were treated for 72h with JQ1 (500 nM), Milcilcib (500 nM) or Palbociclib (2 μM) alone and in combinations of JQ1+Milciclib or JQ1+Palbociclib. RNA was then isolated with TRIzol (Invitrogen, Life Technologies) followed by purification using RNeasy Mini Kit (Qiagen). 50 ng of RNA was used for cDNA synthesis with the Superscript VILO cDNA Synthesis kit (Invitrogen, Life Technilogies). qRT-PCR was performed using SYBR Green. MYCN expression was normalized to Gapdh and analysed as ΔΔCT. Error bars indicate standard deviation of ΔΔCT.

Primer sequences:

| MYCN  (mouse and human) | Forward | AAGAACCCAGACCTCGAGTTTGAC |
| --- | --- | --- |
|  | Reverse | GCAGCAGCTCAAACTTCTTCCAGA |
| Gapdh  (mouse) | Forward | catggccttccgtgttccta |
|  | Reverse | atacttggcaggtttctccagg |

**Animal guidelines**

All animal studies were performed in accordance with protocol (C5/11, C144/13 and C128/15) approval by the Regional Ethical Review Board in Uppsala, Sweden. 6-8 week nude athymic females were purchased from Harlan Laboratories and housed at Uppsala University. Mice had paper houses, paper bedding as cage enrichment, and had access to food and water ad libitum. Post orthotopic transplantation mice were monitored for symptoms of pain and distress the first week. Weight was monitored throughout the study up until the day of sacrifice.
